# Unboxing the T‐box riboswitches—A glimpse into multivalent and multimodal RNA–RNA interactions

**DOI:** 10.1002/wrna.1600

**Published:** 2020-07-06

**Authors:** Jinwei Zhang

**Affiliations:** ^1^ Laboratory of Molecular Biology National Institute of Diabetes and Digestive and Kidney Diseases Bethesda Maryland USA

**Keywords:** riboswitch, RNA interactions, RNA structure, transcription, tRNA

## Abstract

The T‐box riboswitches are widespread bacterial noncoding RNAs that directly bind specific tRNAs, sense aminoacylation on bound tRNAs, and switch conformations to control amino‐acid metabolism and to maintain nutritional homeostasis. The core mechanisms of tRNA recognition, amino acid sensing, and conformational switching by the T‐boxes have been recently elucidated, providing a wealth of new insights into multivalent and multimodal RNA–RNA interactions. This review dissects the structures and tRNA‐recognition mechanisms by the Stem I, Stem II, and Discriminator domains, which collectively compose the T‐box riboswitches. It further compares and contrasts the two classes of T‐boxes that regulate transcription and translation, respectively, and integrates recent findings to derive general themes, trends, and insights into complex RNA–RNA interactions. Specifically, the T‐box paradigm reveals that noncoding RNAs can interact with each other through multiple coordinated contacts, concatenation of stacked helices, and mutually induced fit. Numerous tertiary contacts, especially those emanating from strings of single‐stranded purines, act in concert to reinforce long‐range base‐pairing and stacking interactions. These coordinated, mixed‐mode contacts allow the T‐box RNA to sterically sense aminoacylation on the tRNA using a bipartite steric sieve, and to couple this readout to a conformational switch mediated by tRNA‐T‐box stacking. Together, the insights gleaned from the T‐box riboswitches inform investigations into other complex RNA structures and assemblies, development of T‐box‐targeted antimicrobials, and may inspire design and engineering of novel RNA sensors, regulators, and interfaces.

This article is categorized under:

RNA Structure and Dynamics > RNA Structure, Dynamics and Chemistry
Regulatory RNAs/RNAi/Riboswitches > Regulatory RNAs
Regulatory RNAs/RNAi/Riboswitches > Riboswitches

## Graphical abstract

Recent elucidation of the structures and mechanisms of bacterial T‐box riboswitches provides insights into how structured noncoding RNAs interact with each other and regulate gene expression through multivalent contacts that go much beyond base pairing.

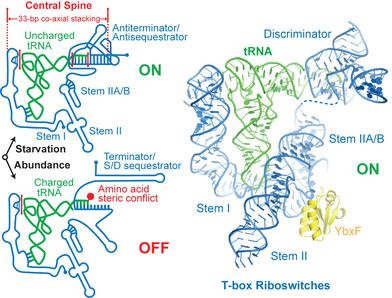

## 1 INTRODUCTION

With the advent of powerful new technologies anchored by next‐generation sequencing, our understanding of the noncoding transcriptome has experienced rapid, sustained growth in the past two decades (Cech & Steitz, 2014). It is increasingly clear that complex RNA–RNA interactions that involve their structures in addition to sequences are major drivers of numerous cellular processes (Dethoff et al., 2018; Langdon et al., 2018; Van Treeck & Parker, 2018). In stark contrast, our appreciation of the RNA structurome and RNA–RNA interactome has remained relatively primitive (Lu et al., 2016; Nguyen et al., 2016). It remains intensely debated to what extent the coding and noncoding RNAs are structured at the secondary, tertiary, and quaternary levels, and their functional consequences. Furthermore, the range, energetics, and kinetics of RNA conformational transformations have only begun to be assessed. Without a quantitative understanding of individual noncoding RNA structures and dynamics, it is even more challenging to characterize the molecular interactions between these dynamic molecules.

Our deficiency in understanding complex RNA structures and interactions are largely due to the limited set of structurally tractable systems consisting of mostly RNA. Among the handful of structurally elucidated ribonucleoprotein systems such as the ribosome and spliceosome, key RNA‐binding proteins play pivotal roles in RNA assembly and interactions (Ramakrishnan, 2014; Shi, 2017). Thus, these ribonucleoprotein assemblies have provided limited knowledge into how large, RNA‐centric or RNA‐only structures such as long noncoding RNAs are architecturally organized, locally and globally folded, and their conformational dynamics and interactions.

The T‐box riboswitches represent a rare tractable paradigm where gene regulation is accomplished through interactions between two structured noncoding RNAs, namely, a T‐box mRNA and a tRNA ligand (Grundy & Henkin, 1993; Putzer, Condon, Brechemier‐Baey, Brito, & Grunberg‐Manago, 2002; Suddala & Zhang, 2019a; Zhang & Ferré‐D'Amaré, 2015). As such, these unique RNA switches provide a treasure trove of information regarding how structured RNAs recognize each other with specificity, affinity, and multivalency. These insights will in turn inform our understanding of other complex RNA assemblies such as long noncoding RNAs, RNA condensates, viral RNA genomes, and so on (Dethoff et al., 2018; Langdon et al., 2018; Van Treeck & Parker, 2018).

## 2 ANATOMY AND ARCHETYPES OF T‐BOX RIBOSWITCHES

**FIGURE 1:**
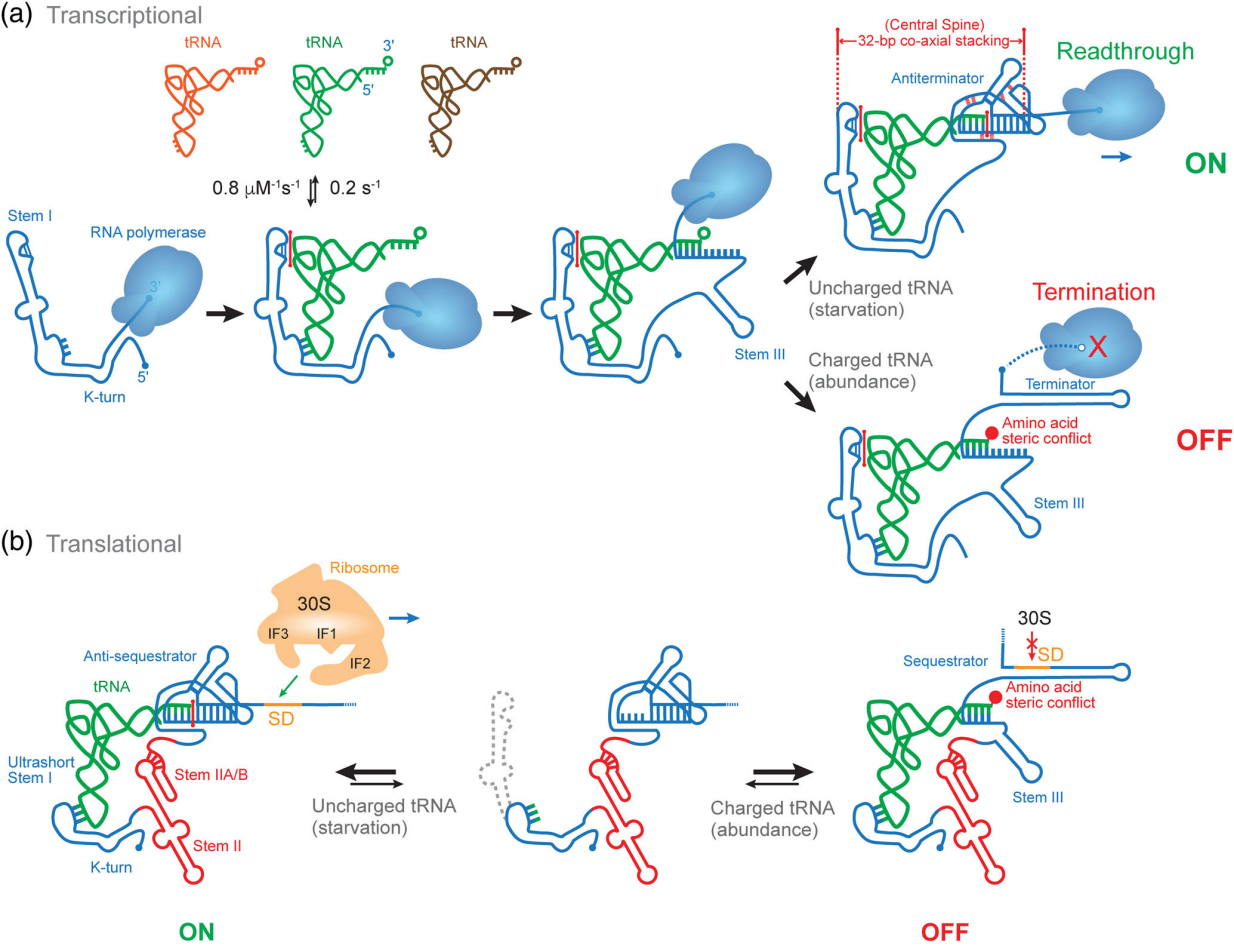
Two classes of T‐box riboswitches. (a) Transcriptional T‐boxes initially bind their cognate tRNAs co‐transcriptionally and bifurcate into two mutually exclusive conformations depending on the aminoacylation state of the bound tRNA. In starvation (upper right), an uncharged tRNA is enveloped by the T‐box mRNA through three distant contacts, producing a continuously stacked “central spine” that stabilizes the transcription antiterminator—allowing gene expression. In nutritional abundance (lower right), the aminoacyl moiety of charged tRNA creates steric conflict with the antiterminator, imploding the structure to form the terminator hairpin shuttering transcription. Red sticks represent intermolecular stacking. (b) Translational T‐boxes are conformationally bistable and can bind uncharged (left) or charged tRNA (right). The former stabilizes the anti‐sequestrator and permits access by the 30S ribosome to the Shine‐Dalgarno (SD) sequence—allowing translation initiation. When a charged tRNA binds, the steric conflict from the aminoacyl drives formation of the SD sequestrator—similar to its transcriptional counterpart—masking the SD sequence and disallowing translation initiation

The T‐box riboswitches can be functionally partitioned into two classes based on their distinct points of regulation—transcription or translation (Figure 1; Gutierrez‐Preciado, Henkin, Grundy, Yanofsky, & Merino, 2009; Vitreschak, Mironov, Lyubetsky, & Gelfand, 2008). The vast majority of currently annotated T‐boxes, more than 1,000 in number, act transcriptionally. They couple the readout of tRNA aminoacylation with an RNA conformational switch that leads to either premature termination of transcription or readthrough into downstream coding genes (Figure 1a). A smaller class, mostly identified in Actinobacteria, controls translation. They utilize the same aminoacylation sensing and similar conformational switching mechanisms as their transcriptional counterparts (Sherwood, Grundy, & Henkin, 2015). However, the conformational switch is used to control the accessibility of the Shine‐Dalgarno sequence by the 30S pre‐initiation complex composed of the 30S ribosomal subunit and initiation factors (IF1‐3), and thus the initiation of translation (Figure 1b; Sherwood et al., 2015). Class‐specific features have been identified in their regulatory behavior as well as their sequence and architecture (Gutierrez‐Preciado et al., 2009; Vitreschak et al., 2008). It is presently unknown which class evolved first. However, horizontal gene transfer likely mediated the expansion of the T‐boxes as opposed to convergent evolution, considering the strong similarity in sequence and structure among most T‐boxes (Gutierrez‐Preciado et al., 2009; Naville & Gautheret, 2009; Vitreschak et al., 2008).

**FIGURE 2:**
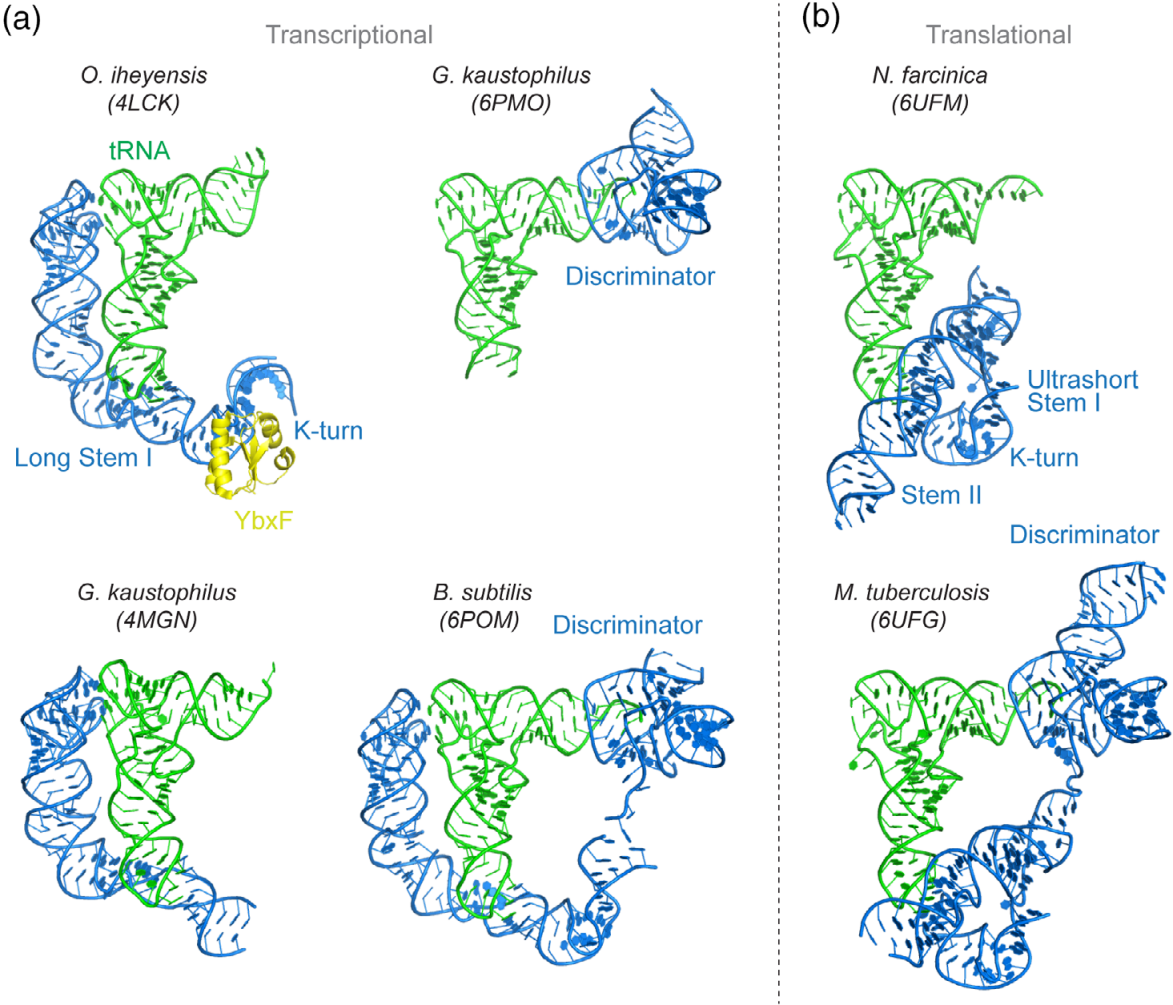
Gallery of T‐box riboswitch‐tRNA complex structures. (a) Transcriptional T‐boxes. Upper left: *Oceanobacillus iheyensis glyQ* T‐box Stem I—tRNA^Gly^—*Bacillus subtilis* YbxF ternary complex (PDB: 4LCK; Zhang & Ferré‐D'Amaré, 2013). Lower left: *Geobacillus kaustophilus glyQ* T‐box Stem I—tRNA^Gly^ complex (PDB: 4MGN; Grigg & Ke, 2013). Upper right: *G. kaustophilus glyQ* T‐box Discriminator—tRNA^Gly^ complex (PDB: 6PMO; Li et al., 2019). Lower right: *B. subtilis glyQS* full‐length T‐box—tRNA^Gly^ complex (PDB: 6POM; Li et al., 2019). T‐boxes and tRNAs are shown in blue and green, respectively, throughout, unless otherwise indicated. K‐turn‐binding protein YbxF is shown in yellow. (b) Translational T‐boxes. Upper: *Nocardia farcinica ileS* T‐box Stem I‐Stem II domains in complex with the cognate tRNA^Ile^ (PDB: 6UFM; Suddala & Zhang, 2019b). Lower: *Mycobacterium tuberculosis ileS* full‐length T‐box in complex with cognate tRNA^Ile^ (PDB: 6UFG; Battaglia et al., 2019)

The T‐box riboswitches are modular RNA devices that consist of an obligate 5′ Stem I domain which decodes the tRNA identity (Grigg & Ke, 2013; Lehmann, Jossinet, & Gautheret, 2013; Zhang & Ferré‐D'Amaré, 2013), an optional intervening Stem II domain which reinforces Stem I‐tRNA interactions (Battaglia, Grigg, & Ke, 2019; Suddala & Zhang, 2019a), and an essential 3′ discriminator domain which senses tRNA aminoacylation and executes genetic switching (Figure 1) (Battaglia et al., 2019; Li et al., 2019; Zhang & Ferré‐D'Amaré, 2014). While the 3′‐most discriminator domain employs a core architecture that is essentially identical among all known T‐boxes, the Stem I and II domains are variable and exhibit interchangeability and characteristics of plug‐and‐play (Figure 2; Gutierrez‐Preciado et al., 2009; Vitreschak et al., 2008). Certain Stem I and II modules are functionally comparable and operationally swappable. The modular nature of the T‐boxes and the distribution of functionally equivalent elements may provide clues into their evolutionary history. This flexibility is consistent with the primary role of the Stem I–II domains being specific recognition of their cognate tRNA ligands (Suddala & Zhang, 2019b). Even with a small molecule ligand, divergent recognition strategies using a range of RNA structural motifs and overall folds have been observed in nature, as exemplified by the six classes of SAM riboswitches that recognize the same small molecule metabolite using diverse folds (Mirihana Arachchilage, Sherlock, Weinberg, & Breaker, 2018; Roth & Breaker, 2009; Sun et al., 2019). Given the significantly increased size and structural complexity of the tRNA ligand compared to small molecules, a plethora of recognition strategies targeting different regions and structural features of tRNA are expected to exist (Table 1). A parallel in the protein world is the manifold strategies used by aminoacyl‐tRNA synthetases (aaRSes; Ibba & Soll, 2000; Terada et al., 2002) and tRNA‐modifying enzymes (Liu, Martinez, Yamashita, & Tomita, 2020; Taniguchi et al., 2018) to recognize their tRNA targets (Naganuma et al., 2014; Zhang & Ferré‐D'Amaré, 2016).

**TABLE 1:** Prominent RNA structural motifs and features in T‐box RNAs and their tRNA ligands

Domain	Name	Structure and Core function	Interacting partner
Stem I	Kink turn (K‐turn)	Architectural element that bends dsRNA trajectory by 120°.	L7Ae superfamily of K‐turn binding proteins. YbxF and YlxQ in *B. subtilis*.
	Specifier	Trinucleotide decoding tRNA anticodon via base‐pairing.	tRNA anticodon
	S‐turn (loop E/bulged G)	Geometric element above specifier avoiding clash with tRNA modification groups.	No known partner
	C‐loop	Flexible junction facilitating tRNA‐induced fit.	No known partner
	Interdigitated double T‐loop motif (IDTM)	Apical element comprising two interlocked pentanucleotide T‐loops producing flat, stackable platforms on both sides.	tRNA elbow (outer corner); T‐loop; D‐loop
Stem II	S‐turn (loop E/bulged G)	Purine‐rich, extensively stacked structural element embedded in dsRNA; laterally stabilizes specifier‐anticodon duplex.	Specifier‐anticodon duplex minor groove
	5‐purine string	Single‐stranded, continuously stacked motif comprising three purines from S‐turn and two adjacent, stacked purines. Forms a triplex with the specifier‐anticodon duplex.	Specifier‐anticodon duplex minor groove
	Inclined tandem A‐minor motif (ITAM)	Recurring motif in which tandem arrays of stacked adenosines interact with dsRNA minor grooves.	Specifier‐anticodon duplex minor groove
	IIA/B pseudoknot	Geometric hub and hinge facilitating docking of stem I with stem II S‐turn. Contains the “F‐box” sequence.	No known partner
Discriminator	Antiterminator	Consisting of helices A1, A2, and an intervening bulge. Conformer that competes with transcriptional terminator hairpin.	tRNA 3′‐NCCA end
	T‐box bulge	A 7‐nt bulge separating helices A1 and A2 and is part of the “T‐box” sequence. The 5′‐tetranucleotide base pairs with the tRNA 3′‐NCCA while the 3′‐ACC trinucleotide stabilizes discriminator structure.	tRNA 3′‐NCCA end; stem III purine string
	Stem III purine string	A 5′‐RRRxG‐stem III‐AA‐3′ motif presents a string of purines along the minor grooves of helices A1 and A2; rejects 2′‐aminoacyl tRNAs.	T‐box bulge; minor grooves of helices A1 and A2
	G•U wobble pair	Terminal base pair of helix A2. Primary steric sensor of 3′‐aminoacyl tRNA. Uridine base clashes directly with a modeled 3′‐aminoacyl.	No known partner
T‐box‐tRNA complex	Central spine	A 32–33 bp continuously stacked helical structure comprising three segments: Stem I IDTM, tRNA T‐arm‐acceptor arm, and antiterminator	Itself an RNA–RNA complex
tRNA	Anticodon	Trinucleotide (nts 34–36) which base pairs with mRNA codon or T‐box stem I specifier. Primary identity element of most tRNAs.	mRNA codon; stem I specifier; stem II S‐turn
	U‐turn	Tetranucleotide motif anchored by U33 of the ASL immediately upstream of the anticodon. Facilitates sharp (180°) turn of RNA backbone. Also present in the TψC‐loop	No known partner
	Anticodon stem loop (ASL)	Stem‐loop (t27–t43) that contains the anticodon, U‐turn, and a 5‐bp stem.	mRNA codon; stem I specifier; stem II S‐turn
	T‐arm (TψC‐arm, TSL)	A stem‐loop structure (t49‐t65) comprising the T‐stem and a pentanucleotide T‐loop motif. ψ is pseudouridine, an isomer of uridine.	D‐loop; stem I IDTM
	D‐arm (D‐loop, DSL)	A stem‐loop structure (t10‐t25) named after its dihydrouridine (D) modification at tU20.	T‐loop; stem I IDTM
	Elbow (outer corner)	Flat, platform structure formed by intercalation between the T‐ and D‐loops. Contains the tG19‐tC56 tertiary pair.	Stem I IDTM
	Acceptor stem	7–9 bp long stem formed by base‐pairing between the 5′‐end and the 3′‐end.	Discriminator
	3′‐NCCA end	Near universal 3′‐end that receives aminoacyl groups for translation or cell‐wall synthesis. N is the discriminator base and one tRNA identity element.	Discriminator

## 3 “DIGITAL” PERCEPTION OF TRNA IDENTITY BY THE STEM I

**FIGURE 3:**
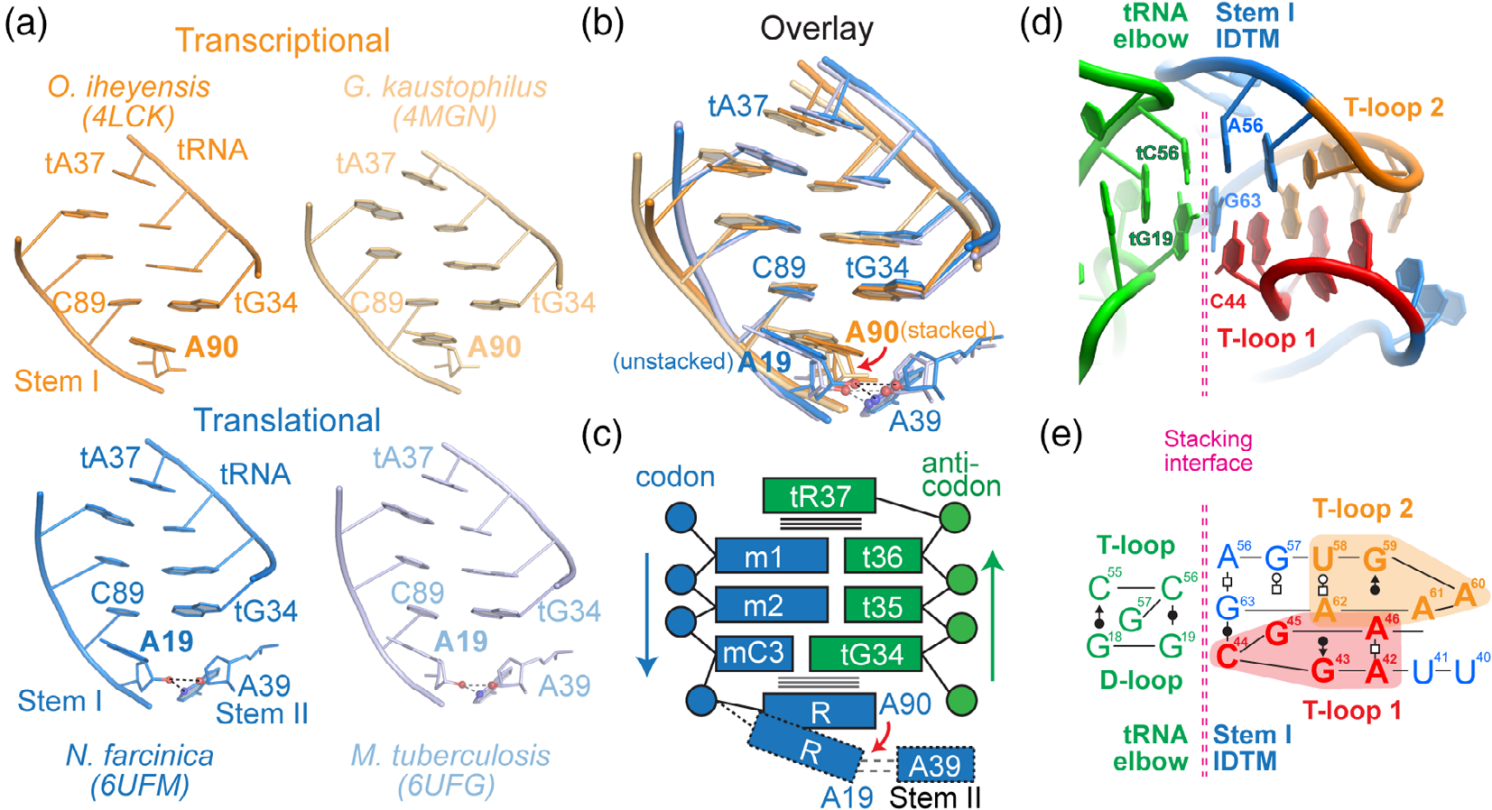
Decoding of tRNA anticodon by the T‐box Stem I. (a) Decoding of the tRNA anticodon by the T‐box specifier codon, illustrated by four co‐crystal structures. (b) Overlay of structures in (a) reveals a positional shift of the conserved purine immediately 3′ of the specifier codon (A90 and A19 in transcriptional and translational T‐boxes, respectively). (c) Scheme of anticodon‐decoding by the T‐box Stem I. (d) Recognition of the tRNA elbow by the Stem I IDTM (Interdigitated Double T‐loop Motif) through platform‐platform stacking interactions (Zhang & Ferré‐D'Amaré, 2013). Interfacial residues are numbered. (e) Anatomy and connectivity of the IDTM in (d). Each of the pentanucleotide T‐loops that interdigitate to form the IDTM is shaded in red and orange, respectively. Noncanonical base‐pairing interactions are illustrated using Leontis–Westhof symbols throughout (Leontis & Westhof, 2001)

A principal variance in T‐box architecture is the choice among long, intermediate, or short versions of Stem I (Gutierrez‐Preciado et al., 2009; Sherwood et al., 2015; Vitreschak et al., 2008). In all known varieties, a codon trinucleotide termed the “specifier” recognizes the anticodon trinucleotide (t34–t36; tRNA numbering preceded by a letter “t”) of its cognate tRNA substrate through three consecutive Watson–Crick base pairs (Figure 3a; Grundy & Henkin, 1993; Zhang & Ferré‐D'Amaré, 2013). This specifier codon can be housed either in a side bulge in long and intermediate Stem I's, or mounted in an apical loop in short Stem I's (Sherwood et al., 2015).

Codon–anticodon interactions are among the most ancient and fundamental RNA–RNA interactions. However, the limited length, thermodynamic stability, and specificity of the three pairs require further stabilization (Grosjean & Westhof, 2016). Peripheral elaborations provide axial, lateral, or both types of reinforcements to buttress otherwise weak codon–anticodon interactions in various biological contexts. The structure of extant tRNA Anticodon‐Stem Loop (ASL) possesses at least two built‐in features that facilitate pairing and stabilization. First, the U‐turn structure (Table 1) enabled by the invariant tU33 of the ASL helps pre‐organize the anticodon into a helical, stacked conformation poised for base‐pairing interactions, improving codon access and reducing the entropic cost of duplex formation (Gutell, Cannone, Konings, & Gautheret, 2000). Second, the conserved purine nucleoside at the t37 position (tR37) naturally following the helical trajectory of the anticodon engage in cross‐strand stacking with the top of the codon–anticodon duplex. To further amplify this helix‐capping effect, tR37 is frequently (70%) posttranscriptionally modified with hydrophobic or bulky moieties such as methylated guanosine (m^1^G; Bystrom & Bjork, 1982), wybutosine (yW; Noma, Kirino, Ikeuchi, & Suzuki, 2006), threonylcarbamoyladenosine (t6A; Miyauchi, Kimura, & Suzuki, 2013) or 2‐methylthio‐N6‐isopentenyladenosine (ms2i6A; Nishimura, 1972), and so on. It is important to note that the chemical elaboration of tR37 was most likely initially driven by the translation process to strengthen tRNA–mRNA interactions, and subsequently co‐opted by the T‐boxes to function outside of the ribosome. Mirroring tR37 on top, long Stem I's feature a functionally critical purine (A90 in *Oceanobacillus iheyensis glyQ* T‐box) that stacks underneath the codon–anticodon duplex with the wobble base pair involving t34, thus creating a stacking “sandwich” that axially stabilizes the duplex (Figure 3b,c; Zhang & Ferré‐D'Amaré, 2013). On the ribosome, the invariant rC1400 (ribosomal RNA numbering preceded by letter “r”) occupies the same position as A90 in the T‐boxes (Korostelev, Trakhanov, Laurberg, & Noller, 2006; Ogle et al., 2001). This tR36‐duplex‐R90 sandwich constitutes an interaction cassette that provides the first stable, specific contact between tRNA and the T‐box RNA. Switching the T‐box specifier allows a different tRNA to activate the mutant T‐box, but never achieving full activity, suggesting other sources of specificity resulting from additional contacts (Grundy & Henkin, 1993).

Indeed, upon establishment of the initial contact between the tRNA anticodon and T‐box specifier codon, additional interactions act to boost overall rates of binding (by 2–20 fold) and prevent premature tRNA release (Suddala et al., 2018; Zhang et al., 2018; Zhang & Ferré‐D'Amaré, 2013). In most T‐boxes, the Stem I is elongated and features an unusual structural element on its distal end (or apex) that recognizes the elbow of the tRNA. Two pentanucleotide T‐loops of the consensus sequences AGAGA and UGxRA (“x” denotes any nucleotide) interlock with each other to form the interdigitated double T‐loop motif (IDTM, Table 1) (Krasilnikov & Mondragon, 2003; Lehmann et al., 2013; Zhang & Ferré‐D'Amaré, 2013). The IDTM is extensively paired and stacked, featuring two central base triples in its core, one solvent‐exposed base triple and six consecutive layers of stacking (each “layer” refers to a base plane; Figure 3d,e). Interestingly, this lateral, six‐layered stack runs perpendicular to the helical base stack of Stem I which it caps, and creates flat, stackable surfaces on both sides of the motif. This is reminiscent of its tRNA ligand, in which two orthogonal flows of stacking converge to form the elbow (Table 1). Thus, the distal IDTM motif is geometrically form‐fitting to interact with the tRNA at the elbow. This strategy of using two interlocked T‐loops to recognize the tRNA elbow (which contains an eponymous T‐loop of its own) is so effective that it is reused at least two other times in the ribosome E site and RNase P, in a remarkable case of convergent evolution (Korostelev et al., 2006; Ogle et al., 2001; Reiter et al., 2010; Zhang & Ferré‐D'Amaré, 2013). Disruption of the IDTM reduced tRNA binding affinity by up to 60‐fold (*K*_D_ increased from 150 nM to 9 μM), association rates by 2‐ to 20‐ fold (*k*_on_ decreased from 0.5 to 0.024 μM^−1^ s^−1^), and abolished tRNA‐mediated transcription readthrough in vivo (Suddala et al., 2018; Winkler, Grundy, Murphy, & Henkin, 2001; Zhang et al., 2018; Zhang & Ferré‐D'Amaré, 2013). Despite its clear role in tRNA binding affinity, it remains unclear how much the elbow interaction contributes to tRNA selectivity.

On the opposite, proximal end of Stem I, a conserved K‐turn motif (or GA motif, Table 1) present in nearly all T‐boxes executes a sharp 120° bend to the dsRNA trajectory (Figure 2a; Lilley, 2014; Winkler et al., 2001; Zhang & Ferré‐D'Amaré, 2013). Without this redirection, the Stem II and discriminator domains stand to extend away from the bound tRNA and cannot make additional contacts required for amino acid sensing. Interestingly, the K‐turn plays essential yet distinct geometric roles of either positioning Stem II (when present), or projecting the discriminator (when Stem II is absent) while occupying essentially the same spatial location near the base of Stem I (Li et al., 2019; Suddala & Zhang, 2019b; Zhang & Ferré‐D'Amaré, 2013). The difference is illustrated by the fact that while the binding of long Stem I to tRNA does not involve the K‐turn, the K‐turn is essential for the short Stem I‐Stem II conjugate to bind tRNA (Li et al., 2019; Suddala & Zhang, 2019b). Interestingly, L7Ae‐family K‐turn binding proteins, such as YbxF in *Bacillus subtilis*, may contribute to the T‐box mechanism by stabilizing the sharply bent conformation of the K‐turn, especially when suboptimal K‐turn sequences are employed or when local intracellular solute conditions including Mg^2+^ concentrations do not sufficiently drive spontaneous K‐turn folding (Baird, Zhang, Hamma, & Ferre‐D'Amare, 2012). It will be interesting to see if any T‐box can actually function without a K‐turn, as proposed for the *Staphylococcus aureus ileS* T‐box (Grundy et al., 1997). It is at least conceptually plausible, especially with the glycine T‐boxes where the Stem I K‐turn is followed by a flexible linker. With Stem II‐containing T‐boxes, the K‐turn is likely required to properly position Stem II, as the latter lacks any sequence complementarity to find its docking site on Stem I unassisted.

## 4 THE MISSING LINK IN THE MIDDLE—UNEXPECTED ROLES OF THE STEM II DOMAIN

In most T‐boxes, the K‐turn module connects Stem I to the enigmatic Stem II domain. This domain is the last principal T‐box structural element whose structure and function was only recently elucidated, due to its significant variability and absence from the well‐studied glycyl T‐boxes (Battaglia et al., 2019; Suddala & Zhang, 2019b). Situated in between the 5′ Stem I and 3′ discriminator domains, the length of the Stem II region ranges from ~10 to 200 nucleotides (nts). A typical Stem II domain, exemplified by the *Nocardia farcinica ileS* T‐box, is about 70 nts long. It contains an elongated Stem II bearing a conserved S‐turn motif (Table 1) near its mid‐section, followed by a so‐called Stem IIA/B element that forms a compact pseudoknot (Figure 1b). This secondary structure arrangement was established nearly two decades ago based on strong phylogenetic conservation among Stem II‐containing T‐boxes (Gutierrez‐Preciado et al., 2009; Vitreschak et al., 2008). However, the tertiary structure and function of the domain have remained elusive. In vitro tRNA‐binding studies clearly showed that Stem II contributed to tRNA binding (Sherwood, Frandsen, Grundy, & Henkin, 2018; Suddala & Zhang, 2019b). In particular, Stem II domain becomes essential when Stem I is naturally truncated in Actinobacteria, such that the critical IDTM motif that binds the tRNA elbow is absent. A logical proposal then followed that Stem II domain is functionally equivalent to the IDTM and may even contact the same general tRNA elbow region. Notably, such a contact, if true, must also be compatible with the IDTM, since most transcriptional T‐boxes contain both a long, IDTM‐containing Stem I together with the Stem II domain. UV‐crosslinking analyses suggested that Stem II domain makes two direct contacts to the tRNA elbow region, to compensate for the absence of the IDTM–elbow interaction (Sherwood et al., 2018).

Surprisingly, two recent cocrystal structures and attendant functional analyses revealed that Stem II domain does not directly contact the tRNA elbow region as proposed (Figure 2b; Battaglia et al., 2019; Suddala & Zhang, 2019b). Instead, the primary function of Stem II domain is to laterally stabilize otherwise weak Stem I codon–tRNA anticodon interactions from the side. This stabilization complements the axial stabilizations from stacking on top and bottom conferred by the Stem I stacking sandwich (Figure 3c). As a result, Stem II partially alleviates the requirements of the stacking sandwich. The structure of the *N. farcinica ileS* T‐box showed that A19 exhibited reduced stacking underneath the codon–anticodon duplex compared to the equivalent A90 in *O. iheyensis glyQ* T‐box (Suddala & Zhang, 2019b). Congruent with this structural observation, an A19U substitution in *N. farcinica ileS* had a mere 2.4‐fold defect while the equivalent A102U in *B. subtilis glyQS* completely abrogated tRNA binding (Suddala & Zhang, 2019b; Zhang & Ferré‐D'Amaré, 2013). Consistent with this, in the *Mycobacterium tuberculosis ileS* T‐box, A19 swings even further away from the codon–anticodon duplex than in *N. farcinica* and appears unstacked (Figure 3; Battaglia et al., 2019). In both *ileS* structures, the rotation of A19 reduces its stacking underneath the C18‐tG34 pair, but acquires a novel bifurcated hydrogen bond from its 2′‐OH to both the N3 and 2′‐OH of A39 of the Stem II S‐turn. The involvement of N3 in this interaction likely drove the selection of a purine at the A39 position in *ileS*, while in *glyQ* the enhanced stacking from the bicyclic purine nucleobase led to a similar purine preference at R90. Obviously, a purine preference could have also resulted from the need to pair with a uridine. Before structures were available, it was thought that the purine preference for A90 in *glyQ* and other long Stem I's was a result of a fourth base pair with the invariant tU33 (Yousef, Grundy, & Henkin, 2005). A recent systematic analysis of single substitutions in the region confirmed that stacking by R90, not pairing with tU33, is the bona fide reason for its conservation (Caserta, Liu, Grundy, & Henkin, 2015). Together, this analysis provides an example where distinct functional contexts converged in the retention of the same RNA side chain pattern—in this case, a clear purine preference. Conceivably, a purine preference can also be driven by their shared N7 group, an excellent hydrogen‐bond acceptor and frequent divalent metal‐binding site. Since purine preferences are easily detectable and frequently encountered in conservation analyses of numerous noncoding RNAs of unknown structure and function, one might form informed hypotheses regarding its underlying drivers: Stacking with the nearest neighbor, pairing with a uridine, hydrogen bonding to N3 or N7, divalent metal binding, and possibly other reasons.

### 4.1 The stem II S‐turn and its 5‐purine string

**FIGURE 4:**
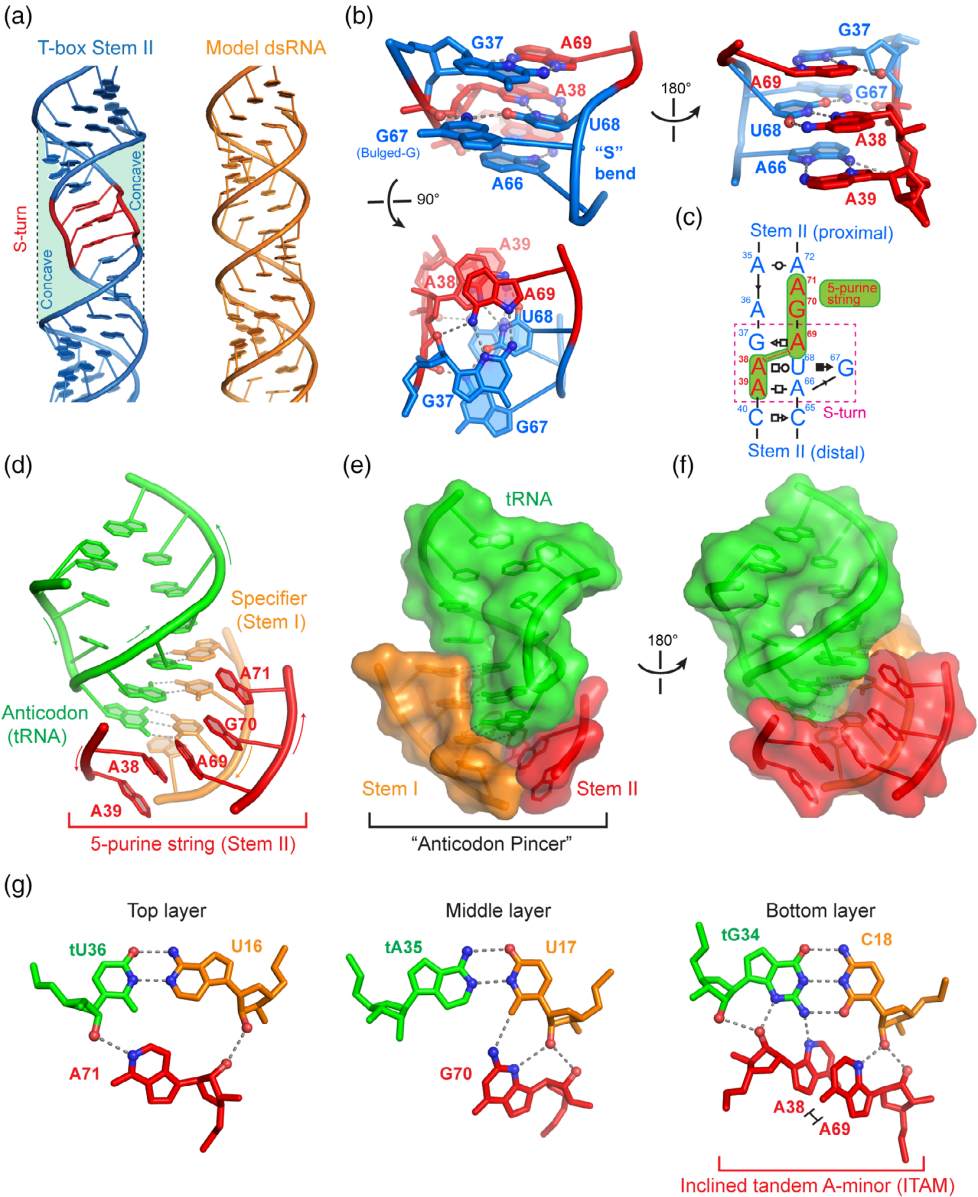
Stabilization of codon–anticodon interactions by Stem II. (a) Widened, irregular grooves of the S‐turn region of *N. farcinica ileS* T‐box stem II (PDB: 4UFM) compared to a model dsRNA (“ideal” dsRNA generated by Coot). (b) Three views of the S‐turn motif in the *N. farcinica ileS* T‐box Stem II. Red residues are those that face and latch the codon–anticodon duplex and form part of a 5‐purine string. (c) Anatomy of the Stem II S‐turn region. The 5‐purine string is shaded in green and the S‐turn boxed. (d) Stabilization of codon–anticodon interactions by the 5‐purine string. Other portions of Stem II are omitted for clarity. (e, f) Two views of the “anticodon pincer” formed by the Stem I codon and Stem II 5‐purine string. (g) Hydrogen bonds between the 5‐purine string and the minor groove of the codon–anticodon duplex at each of the three layers. A38 and A69 cross‐strand stack to form the inclined tandem A‐minor (ITAM) motif

The chief functional element in the Stem II domain is the conserved S‐turn motif located in its middle section. Also known as the loop E or bulged‐G motif, the S‐turn is characterized by two consecutive sharp bends in its backbone producing a namesake “S” shape (Correll, Freeborn, Moore, & Steitz, 1997; Leontis & Westhof, 1998). Unlike the K‐turn or the U‐turn motifs, which bend the RNA path by 120° and 180°, respectively (Table 1), the S‐turn does not cause a bend in the RNA trajectory, and is a helical element frequently embedded in regular RNA duplexes (Yang, Gerczei, Glover, & Correll, 2001). In diametrical opposition to its usual depiction in secondary structure drawings as a bulky, engorged internal loop that appears to bulge out of the duplex on both sides, the S‐turn is actually thinner than the regular dsRNA duplex, and features two wide, recessed grooves (Figure 4a). Indeed, both concaves are used for molecular interactions with proteins or other RNAs. The S‐turn is composed of three stacked layers of mostly purine nucleotides (Figure 4b), and its folding is primarily driven by robust stacking within the motif bolstered by several cation–π interactions (Leontis & Westhof, 1998; Westhof & Fritsch, 2000). Such interactions occur between the two amino groups of G37 and G67 and their adjacent layers (Figure 4b lower panel). The central layer of the S‐turn features a G‐U platform conjugated with a coplanar Hoogsteen A•U pair forming a base triple. The base triple is sandwiched by a sheared G•A pair on top and an A•A pair below, completing a stable structure. The guanosine in the G‐U platform is extruded out of the helical stack forming a small bulge, thus giving the name bulged‐G motif (Figure 4c). However, the G is apparently not required for this structural motif to form, as a G67U substitution produced only a 2.4× defect in tRNA binding (Suddala & Zhang, 2019b). This is consistent with the structure that it is the side opposite the bulged‐G that is used for interaction. On the functional side of the S‐turn, a string of five consecutively stacked purines, A39‐A38‐A69‐G70‐A71, closely tracks the minor groove of the codon–anticodon duplex, laterally stabilizing it (Figure 4d). This 5‐purine string is assembled by the crucial cross‐strand stacking between A38 and A69, which connects the upper trinucleotide with the lower dinucleotide in opposite strands. This cross‐strand stacking in conjunction with the under‐twisted structure of the S‐turn create this linear, extensively stacked string, which essentially forms a third strand with the codon–anticodon duplex forming a triplex‐like structure (Figure 4d–f; Suddala & Zhang, 2019b).

This 5‐purine string carries out three separable functions using its upper, middle, and lower sections. In the upper portion, the A69‐G70‐A71 trinucleotide tilts slightly towards the backside (sugar edge) of the Stem I codon (Figure 4d). Through formation of an extended ribose zipper, A69‐G70‐A71 appears to track and guide the codon trinucleotide A16‐U17‐C18 into a helical, stacked conformation (Suddala & Zhang, 2019b). If true, the purine string would serve to pre‐organize the T‐box codon in anticipation of the tRNA anticodon, reducing the entropic cost of binding. In the middle section, the cross‐strand stacked A38 and A69 form a newly defined motif termed inclined tandem A‐minor (ITAM) motif, which spans and stabilizes the third codon–anticodon pair between C18 and tG34 via 5 hydrogen bonds (Figure 4g). The ITAM is the functional centerpiece of the entire Stem II module and substitution of either adenosine with uridine (A38U or A69U) caused more than 1,000 fold reduction in tRNA binding (Suddala & Zhang, 2019b). These mutations not only disrupt the hydrogen bonds involving ITAM, but would also sever the 5‐purine string near its center, thus disabling the Stem II function in its entirety. At the lower end of the purine string, A39 caps the string and also makes bifurcated hydrogen bonds to A19 of Stem I as discussed above (Figure 3a–c). Together, a network of hydrogen bonds involving 2′‐OH and purine N3 groups form an extended ribose zipper that allows Stem I and II to dock, ultimately creating a binding groove that seems at least partially pre‐configured to bind the specific tRNA anticodon (Figure 4e–f; Battaglia et al., 2019; Suddala & Zhang, 2019b).

### 4.2 The stem IIA/B pseudoknot as a geometric hub

The S‐turn‐bearing Stem II is followed by a Stem IIA/B element forming a 21‐nt compact pseudoknot. The pseudoknot stacks coaxially with Stem II, displacing the base of Stem I which would otherwise stack with the base of Stem II. Both *N. farcinica* and *M. tuberculosis ileS* structures show that the Stem IIA/B pseudoknot makes no direct contact with the tRNA (Battaglia et al., 2019; Suddala & Zhang, 2019b). Nonetheless, it is required for tRNA binding. Deletion of the pseudoknot reduced tRNA binding by ~1,200 fold, 200 fold of which can be recovered by substituting the pseudoknot with an RNA hairpin. This implies that the flat base of the pseudoknot facing Stem II is primarily responsible for facilitating tRNA binding and the entire topology of the pseudoknot is not required (Suddala & Zhang, 2019b). One intriguing proposal is that the pseudoknot appears to be a geometric hub that organizes the docking of Stems I and II and prevents their end‐to‐end stacking. The other side of the pseudoknot—facing the discriminator—could act as a positioning device which brings a string of critical purines 5′ to the Stem III to interact with the antiterminator. Further experiments are required to establish the precise role played by the pseudoknot.

The structural and functional elucidation of Stem II domain resolves several long‐standing mysteries in the T‐box mechanism. First, it explains why the glycine‐specific *glyQ* T‐boxes naturally lack and can function without the Stem II domain (Grundy, Winkler, & Henkin, 2002). A straightforward explanation is that glycine T‐boxes bind their cognate tRNA^Gly^ pairing the former's GGC codon with the latter's GCC anticodon, forming an extensively paired and robustly stacked 3‐bp duplex. This heteroduplex is further axially stabilized by tR37 on top and R90 from below. The strong codon–anticodon interactions likely obviate the requirement for additional lateral stabilization from Stem II, which is only essential for A‐U‐rich codons (Suddala & Zhang, 2019b). Second, it provides a structural basis of the long‐recognized “C rule” of codon usage by the T‐boxes, which states that the third position of the T‐box specifier (codon) is overwhelmingly a cytosine (Gutierrez‐Preciado et al., 2009; Kreuzer & Henkin, 2018; Vitreschak et al., 2008). Comparative sequence analyses showed that this cytosine preference does not correlate with tRNA isoacceptor abundance nor general codon usage on the ribosome (Gutierrez‐Preciado et al., 2009; Vitreschak et al., 2008). Both Stem II‐containing co‐crystal structures revealed that the C rule is imposed by the local structure of the S‐turn ITAM interactions. Specifically, A38 of the ITAM makes two base‐specific hydrogen bonds to both the exocyclic N2 and endocyclic N3 groups of tG34 (Figure 4g, right panel). This uniquely specifies a preference for guanosine in the tRNA anticodon, and indirectly a cytosine in the third position of the T‐box codon (Battaglia et al., 2019; Suddala & Zhang, 2019b). Third, it clarifies the basis of the conservation of the so‐called “F‐box” (Rollins, Grundy, & Henkin, 1997). The conserved nucleotides of the F‐box (^87^CCGUCA^92^, *N. farcinica* numbering) are in fact structural components of the Stem IIA/B pseudoknot facing Stems I and II, forming a key feature required for tRNA binding (Suddala & Zhang, 2019b).

Taken together, specific recognition of the tRNA identity by the Stem I and II domains sets the stage for the T‐box discriminator domain to probe the tRNA 3′‐end to check for aminoacylation, and to couple this readout with an RNA conformational change that governs downstream gene expression.

## 5 SENSING AND RESPONDING TO TRNA AMINOACYLATION

The most essential and unique feature of the T‐box mechanism is the ability to sense the presence or absence of an esterified amino acid on the tRNA 3′‐end and regulate downstream gene expression accordingly. This is no easy task and unprecedented for a small RNA‐only device, which comprises only about 60 nts. This function is executed by a highly compact structure termed the T‐box discriminator, and was only recently defined based on its structural and functional elucidation (Li et al., 2019; Weaver & Serganov, 2019). It is composed of three helices, namely, Stem III, Helix A1, and Helix A2, connected by three crucial single‐stranded RNA (ssRNA) elements that precede each helical component (Li et al., 2019). In principle, to construct an effective sensor and responder of tRNA aminoacylation, the following four “skills” are required: (a) an ability to capture the tRNA 3′‐end from an ocean of competing RNAs, (b) an ability to judge if an amino acid is present, (c) a bistable output system that executes genetic switching, and (d) a coupling mechanism that binds each sensory readout (input state; amino acid present or absent) with a corresponding regulatory outcome (output state; gene on or off). The following section briefly chronicles how the core mechanisms of sensing and responding to tRNA aminoacylation were conceptually developed, biochemically defined, and ultimately structurally validated.

### 5.1 T‐boxes are bona fide RNA sensors of tRNA aminoacylation

Pioneering genetic and biochemical work established that in addition to the three codon–anticodon base pairs from Stem I, a second set of base‐pairing interactions form between the tRNA 3′‐NCCA terminus and the so‐called T‐box bulge (Table 1; Rollins et al., 1997; Yousef, Grundy, & Henkin, 2003). It was initially thought that T‐boxes reject charged tRNAs simply because the aminoacyl group interferes with the formation of these four base pairs so that the tRNA 3′‐end cannot bind to the T‐box bulge. However, spatial considerations suggested that an amino acid esterified to the ribose of the terminal tA76 is on the opposite side of and points away from the base stack, and thus cannot directly interfere with base pairing. Further, while the in vivo data was compelling, it was not demonstrated using biochemically defined components that the T‐box RNA can indeed sense tRNA aminoacylation without the help from proteins known to possesses this ability such as EF‐Tu (Janiak et al., 1990; LaRiviere, Wolfson, & Uhlenbeck, 2001). Instead of true aminoacyl‐tRNAs, a charged tRNA mimic was used—a tRNA that carries an additional cytosine on its 3′‐end (3′‐NCCAC, termed Ex1C for “extra” cytosine) (Yousef et al., 2003, 2005). Indeed, the experiments using charged tRNAs are technically challenging, due to the difficulty in obtaining an adequate amount of sufficiently pure aminoacyl‐tRNAs and the hydrolytic instability of the aminoacyl linkage. To overcome this problem, a page was borrowed from the chemical biology books. Using the flexizyme—an in vitro selected aminoacylating ribozyme (Goto, Katoh, & Suga, 2011; Xiao, Murakami, Suga, & Ferre‐D'Amare, 2008)—to aminoacylate and reversible N‐pentenoyl protection of the aminoacyl group to purify charged tRNAs (Lodder, Wang, & Hecht, 2005), a new, flexible method was developed to produce biophysical quantities of essentially any charged tRNA to >95% purity (Zhang & Ferré‐D'Amaré, 2014a). This allowed for the first demonstration that the T‐box RNA itself is able to sense and respond to tRNA aminoacylation in vitro, making it a true “ribo”switch (Zhang & Ferré‐D'Amaré, 2014).

With the ability to sense tRNA aminoacylation confirmed, the crux of the problem became how an RNA can read a minute chemical change on another RNA. Using a series of tRNA variants that carry diverse chemical adducts to the ribose of tA76, it was demonstrated using *B. subtilis glyQ* T‐box that the molecular volume of the tRNA 3′ group is the best predictor of the tRNA‐mediated transcription readthrough (Zhang & Ferré‐D'Amaré, 2014). The larger the tRNA 3′ moiety, the lesser the readthrough—suggesting a steric occlusion mechanism is at play. Importantly, a 3′‐glycine already elicits the full rejection by the T‐box and making it larger does not further reduce readthrough transcription. This suggests that the steric rejection threshold is set at or less than the size of glycine—the smallest amino acid—thus allowing the same discriminator architecture to sense and reject all aminoacyl‐tRNAs. Notably, the T‐box is an “ON” switch whose true cognate ligand is an uncharged tRNA of a prescribed specificity. It selects for the characteristic 2′,3′‐*cis* diols present on the RNA 3′‐end, does not distinguish among the amino acids and reject them blanketly. It also rejects other chemical moieties that are not amino acids, such as azido and phosphate groups, suggesting a general steric sieve that is intolerant of any significant bulk larger than ~60 Å^3^ at the tRNA 3′‐end (Zhang & Ferré‐D'Amaré, 2014).

### 5.2 Structural basis of aminoacylation sensing

**FIGURE 5:**
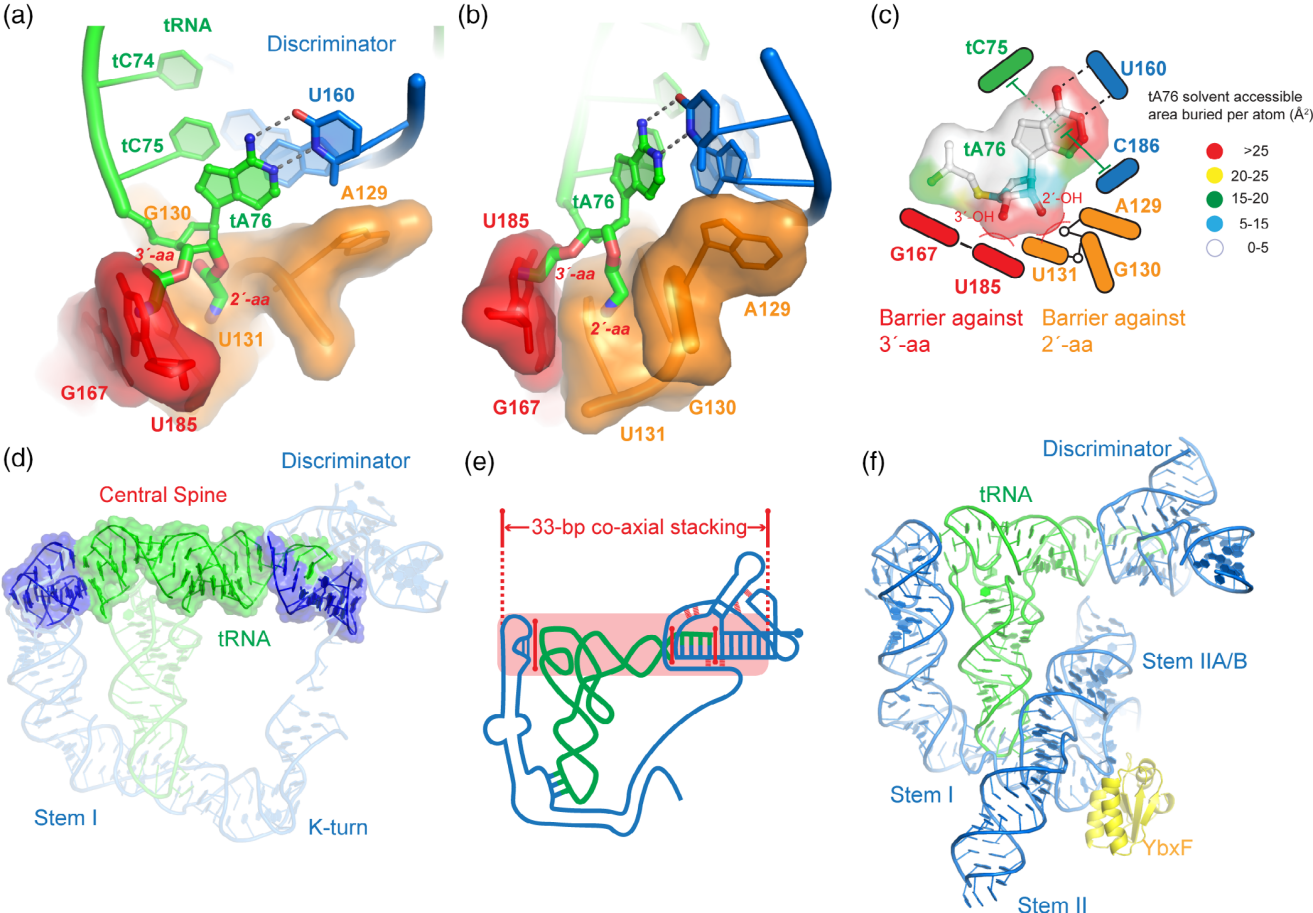
Molecular basis of RNA‐actuated sensing of tRNA aminoacylation and genetic switching. (a, b) Two views of the composite steric sieve against aminoacyl‐tRNAs. Modeled 2′‐ and 3′‐aminoacyl groups are shown in thick sticks. The steric barriers against 2′‐ and 3′‐aminoacyl are shown in surface representations and colored orange and red, respectively. (c) Cartoon scheme of the immobilization and steric selection of uncharged tRNA by a T‐box discriminator. The terminal nucleotide of tRNA, tA76, is colored based on surface burial by the discriminator. (d, e) Cryo‐EM Structure (d) and cartoon scheme (e) of a continuously stacked central spine formed by coaxial stacking of the upper half of tRNA with the Stem I IDTM and Discriminator. Three solid red lines indicate intermolecular coaxial stacking and dotted lines in the discriminator denote tertiary contacts. (f) Simple combination of three tRNA‐bound co‐crystal structures of T‐box Stem I (PDB: 4LCK), Stem II (PDB: 6UFM), and Discriminator (PDB: 6PMO) domains produce a feature‐complete T‐box‐tRNA complex model, which represents the most typical T‐box such as the original *B. subtilis tyrS* T‐box

As is with any steric occlusion mechanism, a snug binding pocket must exist on the T‐box discriminator that fully encompasses the tRNA 3′‐end. This steric pocket is recently visualized by two highly similar co‐crystal structures—one transcriptional and the other translational—and further confirmed by a cryo‐EM structure, covering three different species (Battaglia et al., 2019; Li et al., 2019). A major roadblock that precluded structural analyses was the incorrect demarcation of domain boundary. The prevailing view was that the less conserved Stem III region was part of the variable inter‐domain linker and the antiterminator (consisting of Helices A1 and A2 and a 7‐nt intervening T‐box bulge; “A” denotes “antiterminator”) was sufficient for binding tRNA 3′‐end, sensing aminoacylation, and conformational switching. It was only recently recognized that the Stem III region is an integral part of the 3′‐aminoacylation‐sensing domain, is indispensable for function, and is not part of the linker (Li et al., 2019). Without Stem III, the antiterminator alone was unable to bind tRNA appreciably. RNA structure‐guided sequence alignment revealed hidden sequence conservation on both flanks of Stem III, manifest as a 5′‐RRRxG‐Stem III‐AA‐3′ motif. This highly conserved motif was not recognized previously because it was obscured by the variability of the Stem III stem and loop. Together with another conserved purine at the 5′ edge of Stem III (G133), this string of conserved purines tracks the fused minor grooves of the heteroduplex between the tRNA 3′‐NCCA and T‐box bulge and of Helix A2 (Li et al., 2019). This string of purines forms a triplex with the dsRNA, reminiscent of the 5‐purine string of Stem II S‐turn forming a triplex with the codon–anticodon duplex. Unlike the straight, well‐stacked Stem II purine string, the Stem III purine string is more extended; it bends to follow the minor groove and crosses over Stem III to continue to track the Helix A2 minor groove. Together with the adjacent antiterminator, Stem III and its flanking purines form a compact, single functional unit that was defined as the T‐box “discriminator”, as it is necessary and sufficient to sense tRNA aminoacylation (Li et al., 2019). The discriminator deploys a two‐component steric sieve to select against two classes of aminoacyl‐tRNAs (Figure 5a–c). A conserved wobble base pair (G167•U185) at the base of Helix A2 chiefly rejects amino acids attached to the tRNA 3′‐OH, whereas the purine string, especially the backbone bend near G130, rejects the 2′‐aminoacyl group (Figure 5a–c). Due to rapid regioisomerization between the two forms of aminoacyl‐tRNAs at ~5 s^−1^, it is necessary to guard against both types, to ensure efficacious sensing of tRNA aminoacylation. In conclusion, the new discriminator‐tRNA complex structures not only validated the proposed steric sensing mechanism based on biochemical analyses (Zhang & Ferré‐D'Amaré, 2014), but also revealed the precise composition of the steric sieve and illustrated how it is coupled to the conformational switch.

### 5.3 A tRNA‐actuated mRNA switch

After tRNA aminoacylation is sterically sensed, how is the readout communicated and coupled to genetic switching? In other words, how does the uncharged tRNA specifically stabilize the “ON” conformation of the discriminator? The key lies in an intermolecular coaxial stacking interaction between the tRNA 3′‐end and Helix A1 of the T‐box discriminator. Intramolecular coaxial stacking of adjacent dsRNA helices within a complex RNA molecule is principally responsible for shaping the overall architecture of structured RNAs (Butcher & Pyle, 2011; Walter et al., 1994). Intermolecular stacking between interacting RNAs, however, is much less explored owing to the scarcity of examples. The T‐box system provides a proof‐of‐concept that intermolecular stacking between RNAs is a key driver of binding affinity, specificity, and stability. The original idea of tRNA 3′‐end stacking with the T‐box RNA came about 10 years ago (N. Baird, personal communication) and was inspired by the curious observation that to maintain the binding of tRNA 3′‐NCCA terminus to a randomized 7‐nt T‐box bulge, the UGGN tetranucleotide complementary to the tRNA 3′‐end is free to slide within the bulge (Fauzi, Agyeman, & Hines, 2009). However, in all known T‐boxes, this UGGN sequence is invariably fixed at the 5′ edge of the bulge, that is, immediately adjacent to Helix A1 (Gutierrez‐Preciado et al., 2009; Vitreschak et al., 2008). Thus, the putative intermolecular helix formed between the tRNA 3′‐end and the T‐box bulge must be juxtaposed with Helix A1 with no intervening nucleotides. This led to the hypothesis that the two adjacent helices stack with each other and this stacking allows the tRNA to stabilize Helix A1, and in turn the “ON” conformation of the discriminator. To probe for this stacking interaction, stacking‐quenched nucleoside analogs 2‐aminopurine (2AP) and pyrrolo‐cytosine (PyC) were placed at the tRNA‐T‐box interface on each side, respectively. By quantitatively comparing the extent of fluorescence quenching of 2AP and PyC in full‐length T‐box‐tRNA complexes with a set of benchmarks of known structures assembled using oligonucleotides, clear evidence was obtained that the tRNA 3′‐NCCA terminus not only base‐pairs with the T‐box bulge, but importantly stacks coaxially with Helix A1 leading to its stabilization (Zhang & Ferré‐D'Amaré, 2014). This finding was further confirmed and extended by calorimetry analysis that this tRNA‐Helix A1 stacking contributes ~0.9 kcal/mol of binding energy.

Three independently solved structures eventually validated this model and directly visualized the functionally critical tRNA–Helix A1 stacking interaction (Battaglia et al., 2019; Li et al., 2019; Weaver & Serganov, 2019). Remarkably, this stacking interface (tA76||C186) is immediately adjacent to and covalently linked with the primary steric sieve (G167•U185). This spatial proximity and linkage couples the perceived tRNA aminoacylation state (Y/N) with the corresponding regulatory outcome (OFF/ON), as the tRNA‐Helix A1 stacking is deterministic for the choice between the ON or OFF conformer (Li et al., 2019). The aminoacyl group, if present, sterically clashes with the nucleobase of U185 and pushes it away by ~2 Å. This movement pulls its neighboring C186 away from tA76 with which it normally stacks, and helps shear Helix A1 to form the “OFF” conformer—the terminator hairpin (Li et al., 2019).

Remarkably, the tRNA‐Helix A1 coaxial stack extends further upstream into the tRNA acceptor and T stems (Table 1), out of the elbow and into the 6 stacked layers of the IDTM (Figure 3d,e), creating a continuously stacked, 33‐layered “central spine” (Figure 5d,e). This helical spine is created by the concatenation of four helical segments through serial intermolecular stacking at three interfaces. It is this extended RNA backbone, first proposed based on biochemical characterizations and recently visualized by a 4.9 Å resolution cryo‐EM structure, that provides the stability to lock the discriminator in the “ON” state, temporarily overcoming a much more stable terminator or sequestrator hairpin structure corresponding to the “OFF” state (Li et al., 2019). Importantly, the structure shows that the three principal contacts to the tRNA anticodon, elbow, and 3′‐NCCA terminus are concurrently engaged in the “ON” state. Single‐molecule analysis of the same *B. subtilis* full‐length T‐box complex detected an “ultrastable” complex with the half‐life of about 1 hr, revealing the unusual stability of this multivalent complex (Suddala et al., 2018). Interestingly, deletion of the Stem I IDTM motif that stacks with the tRNA elbow abrogated this stable complex, suggesting that the IDTM‐elbow interaction directly contributes to complex stability. It is less clear how exactly the IDTM achieves this. It could provide additional stabilizing energy by adding six layers of stacking onto the central spine (a thermodynamic effect), or it could maintain the tRNA elbow contact by acting as an anchor to reduce premature tRNA release (a kinetic effect), or both.

Taken together, the original tRNA‐T‐box stacking concept is supported by biophysical characterizations using fluorescence probing and calorimetry, biochemical characterizations employing in vitro termination‐readthrough analyses, and complemented by recent high‐resolution structural data. These findings suggest a potentially general mechanism of gene‐expression control mediated by intermolecular RNA–RNA stacking, and provide a proof‐of‐principle that one structured RNA can act as a trans‐activator for another RNA conformational switch, through a combination of base‐pairing, stacking, and tertiary interactions. Based on this concept, RNA therapeutics can be potentially designed to manipulate conformations of bistable RNA elements to control gene expression, splicing, microRNA targeting, and so on.

## 6 DIFFERENCES BETWEEN TRANSCRIPTIONAL VERSUS TRANSLATIONAL T‐BOXES

A number of differences exist between the two major classes of T‐boxes that regulate transcription and those modulating translation, both in their regulatory behavior as well as sequence and structure (Figure 1). The next section explores how the divergences in RNA sequence and structure may correlate with their distinct regulatory needs in terms of the kinetic and thermodynamic properties of tRNA association, dissociation, and conformational switching.

### 6.1 Differences in regulatory behavior

One predominant difference between the two types of T‐boxes is the distinct kinetic behavior of their regulatory mechanisms. Transcriptional T‐boxes are “one‐off” switches that make a single genetic decision within a short time window which either permits or forbids an elongating RNAP from traversing a “checkpoint” region in DNA. This region encodes a hairpin‐dependent, intrinsic transcription terminator—a strong RNA hairpin followed by a track of uridines—which guards the entrance into the downstream open reading frame (Grundy & Henkin, 2004; Zhang, Lau, & Ferré‐D'Amaré, 2010). Up to this checkpoint, tRNA rapidly binds and dissociates from a growing T‐box mRNA transcript that emerges from the RNA exit channel of the RNA polymerase (RNAP) and folds co‐transcriptionally. This vectorial folding of the T‐box RNA may be further influenced by simultaneous engagement of tRNA or protein factors (i.e., K‐turn‐binding proteins). Subsequently, when the T‐box mRNA reaches a certain length, the bound tRNA becomes immobilized and unable to exchange with tRNAs in solution, when the overall tRNA–mRNA complex stability surpasses a certain threshold (Grundy & Henkin, 2004). At this point, the 3′‐halves of the Helices A1 and A2 (~12 nts long) will attempt to anneal with its 5′ halves, while the intervening T‐box bulge is presumably already paired to the tRNA 3′‐NCCA end. With uncharged tRNAs bound to T‐boxes under starvation conditions, the annealing is successful and the Stem III purine string latches on, stabilizing the stacking between tRNA 3′‐end and Helix A1. This completes the central spine conferring extraordinary stability and precluding terminator formation, turning the gene ON. In amino acid abundance, the 3′‐aminoacyl moiety itself creates steric conflicts with the discriminator, blocking the annealing of the 3′‐strands of Helices A1 and A2, and/or the engagement of the Stem III purine string with the minor groove. Lack of stabilization due to the severed central spine allows the 3′‐strands of Helices A1 and A2 to form the 5′‐strand of an extended terminator hairpin. Together with the slippery uridine track, the hairpin shears the RNA–DNA hybrid and extracts the nascent RNA transcript from the RNAP catalytic center, causing the otherwise highly stable elongation complex to collapse (Peters, Vangeloff, & Landick, 2011). Thus, the transcriptional T‐boxes act shortly after transcription initiation and render an irreversible decision controlling the conditional transit of RNAP from the 5′‐UTR into the ORF, depending upon the charging ratio of their cognate tRNAs. The “ON” and “OFF” states do not interconvert and correspond to chemically distinct RNA species as long, ORF‐bearing and short, prematurely terminated transcripts, respectively.

In stark contrast, translational T‐boxes are bistable RNAs that are chemically homogenous but conformationally distinct. At least two conformational states are in rapid equilibrium, enabling the RNA to continuously monitor the charging levels of specific tRNAs and change conformations accordingly. Here, the “ON” and “OFF” states do interconvert and do not correspond to chemically distinct species, but rather different conformational states of the same RNA. By exposing or sequestering the Shine–Dalgarno sequence to which ribosomal RNA binds, the “ON” and “OFF” conformational states control translation initiation. Regulation does not require active transcription as do the transcriptional T‐boxes. These decidedly different modes of tRNA interaction and structural switching between the two T‐box classes presumably drove the adoption of distinctive sequence and structural features discussed below.

### 6.2 Differences in sequence and structure

A principal difference in the architecture of transcriptional and translational T‐boxes is the length and variability of Stem I. Transcriptional T‐boxes generally require long Stem I's featuring the IDTM motif that binds the tRNA elbow. Mutations that disable the IDTM significantly compromise T‐box function in vitro and in vivo (Suddala et al., 2018; Zhang et al., 2018; Zhang & Ferré‐D'Amaré, 2013). While a few translational T‐boxes also possess the canonical long Stem I's, most translational T‐boxes feature Stem I's that are intermediate or “ultrashort” in length, both lacking the IDTM motif (Sherwood et al., 2015). The general expendability of the IDTM in translational T‐boxes contrasts with its essentiality in their transcriptional counterparts and may indicate a relaxed requirement for tRNA association rates or overall complex stability, or both. Faster association rates are favored by transcriptionally acting riboswitches so that higher ligand occupancy can be achieved by the time when the RNAP reaches the terminator region (Wickiser, Winkler, Breaker, & Crothers, 2005). The genetic decision is frequently made before the ligand‐riboswitch binding reaches equilibrium (Zhang, Lau, & Ferré‐D'Amaré, 2010). For transcriptional T‐boxes, the IDTM accelerates tRNA binding so that reasonable occupancy is achieved before RNAP reaches the terminator, so that the switch can function in a tRNA‐dependent manner (Suddala et al., 2018; Zhang et al., 2018). As the default, tRNA‐free state of the T‐box is “OFF”, the IDTM increases the expression of downstream genes. In translational T‐boxes, there is no strict time limit for tRNA association other than their decay by ribonucleases, since sensing and genetic switching are constantly ongoing. This chief difference in tRNA‐binding kinetics may underlie the strong variance in Stem I. On the other hand, the transcriptional T‐boxes may additionally require higher thermodynamic stability than their translational variants. Translational T‐boxes must balance the relative thermostabilities of the two conformers and exhibit manageable affinities for tRNAs, so that they remain fluid enough between the ON and OFF states and can release and rebind other tRNAs. Notably, while most translational T‐boxes lack the long Stem I's, they frequently sport additional insertions in their discriminator domains, such as in Stem III and especially in Helix A2 (i.e., on top of the antiterminator). These features may aid the folding of the discriminator helical elements and facilitate their refolding between the conformers, or even make additional contacts to the tRNA acceptor stem, as proposed for the Stem Sa in the *Staphylococci glyQ* T‐boxes (Apostolidi et al., 2015; Stamatopoulou et al., 2017).

Importantly, the two classes of T‐boxes share a highly congruent core structure which captures and probes the tRNA 3′‐end and actuates switching via tRNA–mRNA stacking (Battaglia et al., 2019; Li et al., 2019; Zhang & Ferré‐D'Amaré, 2014). This universal sensory and regulatory core is then elaborated with peripheral features that are much more divergent. This mirrors the radial gradient of conservation from inner catalytic centers to outer surfaces found in many protein enzymes, such as the RNAP that synthesized the T‐boxes (Vassylyev et al., 2007; Zhang, Palangat, & Landick, 2010). It is all but certain that the divergent features on the T‐boxes act to modify or fine‐tune the core mechanism. In particular, species‐specific insertions may engage other surfaces and features of the tRNA ligand in novel and potentially specific ways, just like their protein counterparts (Windgassen et al., 2014).

## 7 LESSONS LEARNED FROM THE T‐BOXES

The recent flourish of genetic, biochemical, single‐molecule, and structural analyses into the T‐box riboswitches have shined a timely light on understanding multivalent and multimodal RNA–RNA interactions in general. Three chief conceptual advances gleaned from the T‐box paradigm are discussed below.

First, RNA–RNA interactions can effectively leverage multivalency. The archetypal T‐box system features three RNA–RNA interfaces that are interestingly roughly equidistant from each other at ~60 Å apart (Figure 5d–f). These distant sites are constructed using distinct structural motifs (Table 1), employ unique interaction modalities, and exhibit distinguishable energetic characteristics. The central interface between the T‐box codon and tRNA anticodon is likely the first contact and confers the highest specificity and strength. It involves three Watson–Crick base‐pairs axially reinforced on one or both sides by cross‐strand stacking, and is frequently also laterally reinforced in the minor groove by a 5‐purine‐string presented by the Stem II S‐turn. The first distal interface between the Stem I IDTM and tRNA elbow is largely aromatic stacking in nature involving no base pairs. As such, it lacks the specificity to engage without a prior contact (Zhang & Ferré‐D'Amaré, 2013). The second distal interface between the tRNA termini and the T‐box discriminator consists of four Watson–Crick base pairs, two instances of intermolecular coaxial helical stacking (involving the 5′ tG1 and 3′ tA76, respectively), six sets of minor groove interactions with a bipartite six‐purine string, and finally three nucleobase–ribose packing contacts (Li et al., 2019). The individual affinities and specificities of the three binding sites follow the order of codon–anticodon > tRNA 3′‐end‐discriminator > IDTM‐elbow. The three sites function in concert to construct a remarkably stable tRNA‐T‐box complex that enables downstream gene transcription or translation. Conceivably, the distribution of total binding energy across multiple distant interfaces is advantageous in achieving improved specificity in the selection of the intended targets. Such multivalent binding mode allows for the simultaneous recognition of both the overall shape and dimensions of the target as well as of local features that match the expectations. For example, this system can guard against fortuitous binding by small RNAs that happen to present a complementary sequence to the T‐box codon or the T‐box bulge, and by tRNAs that have the same termini and elbow but mismatched anticodon.

Second, RNA–RNA interactions can employ multi‐segment, concatenated intermolecular stacking. It is well recognized that intramolecular coaxial stacking between dsRNA helices is a chief sculptor for overall RNA architectures (Dussault, Dube, Jacques, Grondin, & Lafontaine, 2017; Walter et al., 1994; Westhof & Fritsch, 2000). The T‐box system extends this notion and reveals that intermolecular RNA–RNA interactions also frequently utilizes stacking in addition to pairing at their interfaces. Long‐range base‐pairing interactions produce short dsRNA helical segments. The hydrophobic ends of the helices have a strong inherent tendency to form coaxial stacks. This tendency is driven by enthalpically favorable π–π interactions and augmented by favorable entropy associated with the liberation of constrained solvent shells surrounding the hydrophobic terminal base planes. When bound to the T‐box, both termini of the tRNA stack with the T‐box discriminator, stabilizing the latter to permit gene expression. On the opposite side, the tertiary base pair of the tRNA elbow stacks with the IDTM base triple and the stacking flows through all six layers of the IDTM. Thus, three intermolecular stacking contacts join together four helical segments spanning 33 contiguous base planes, forming the central spine that provides requisite thermostability to overcome the transcriptional terminator. This natural phenomenon of multiple RNA helices going to great lengths to concatenate and assemble into long, contiguous helices echoes the *in crystallo* formation of pseudo‐infinite helices (Shoffner, Wang, Podell, Cech, & Guo, 2018; Zhang & Ferré‐D'Amaré, 2014b). The latter is a primary driver of nucleic acid crystal formation. We anticipate the existence of many more such coaxially stacked assemblies in long noncoding RNAs and even RNA condensates (Tauber et al., 2020).

Third, RNA–RNA interactions can utilize mutually induced fit. Large RNAs are dynamic, flexible entities and can possesses multiple domains. Just like protein–protein interactions, major conformational changes in both the T‐box and the tRNA accompany and enable their binding. The Stem I hinge near the C‐loop pivots in order to accommodate the flexing of the tRNA t26•t44 hinge. In translational T‐boxes to which tRNAs reversibly bind and dissociate from, the discriminator domain most likely has to reorganize its fold upon tRNA binding and dissociation. In particular, the string of purines that flank Stem III presumably can only bind after its binding site forms, which is the minor groove of the tRNA‐T‐box heteroduplex. Upon tRNA departure, the loss of the positioning contacts will likely lead to dissociation of the Stem III purine string and partial collapse of the discriminator structure. The drastic structural differences between an isolated T‐box antiterminator domain and its tRNA complex lends support to the notion of induced fit by tRNA (Gerdeman, Henkin, & Hines, 2003; Li et al., 2019; Yousef et al., 2005).

These critical themes and insights gathered from the T‐box paradigm are potentially applicable in a wide range of large noncoding RNAs, RNA condensates, and RNP assemblies, and are expected to usher in a new wave of structural and mechanistic elucidations of complex noncoding RNAs.

## 8 OPEN QUESTIONS AND OUTLOOKS

Despite the recent unraveling of the core T‐box mechanism (Weaver & Serganov, 2019), there is still much to decipher in the diverse pool of more than 1,000 annotated T‐boxes. At the time of writing, the simplest *B. subtilis glyQS* T‐box remains the only T‐box that has been successfully reconstituted in vitro with defined components. Intriguingly, the *B. subtilis thrS* T‐box gained function in vitro upon the addition of a partially purified protein fraction, suggesting that there could be additional components in the T‐box regulon (Putzer et al., 2002). In addition, several key mechanistic questions within the T‐box paradigm still await further investigation, especially regarding the T‐box adaptation to tRNA modifications, a detailed kinetic framework of sequential tRNA binding and conformational switching, as well as structures and functions of lineage‐specific features such as tandem T‐boxes, domain insertions and appendages, and other novel elaborations to the core mechanism.

First, it remains unclear to what extent the T‐box sequence and structure have been gradually sculpted by the emergence of numerous post‐transcriptional modifications on their tRNA ligands through their co‐evolution. T‐box riboswitches are ancient gene‐regulatory elements that might have existed in the RNA world considering its functional independence from proteins (Zhang & Ferré‐D'Amaré, 2014). Due to their intimate, multivalent interactions with tRNAs, which are among the most extensively chemically modified biomolecules, the T‐boxes must accommodate or perhaps even proactively recognize these modifications. One example of accommodation is how the Stem I S‐turn region immediately above the specifier‐anticodon duplex tilts away from tRNA, avoiding steric clashes with the heavily modified tR37 (Zhang & Ferré‐D'Amaré, 2013). Chemical biological and structural analyses are needed to determine how T‐boxes accommodate or actively exploit tRNA modifications, potentially for improved affinity and specificity. Another intriguing idea is that since the modification groups themselves are frequently derived from amino acids (e.g., the nearly universal threonylcarbamoyladenosine at tR37), the potential recognition of tRNA modifications by T‐boxes could provide a second checkpoint to monitor amino acid availability, in addition to sensing aminoacylation on the tRNA 3′‐end (J. Alfonso, personal communication). However, it is unclear whether the timing and reversibility of the modifications allow them to act as effective nutritional markers.

Second, we lack a detailed kinetic picture comprising the sequence and timing of a series of discrete binding events between the tRNA and T‐box RNA and of the conformational switching that follows. The intimate, short‐range Stem II S‐turn interactions with the codon–anticodon duplex, despite engaging numerous hydrogen bonds, lack base complementarity and thus the addressing power to locate to its target site. Thus, the S‐turn likely can only serve as a secondary contact and requires spatial guidance by the Stem I, K‐turn and pseudoknot. Considering the 5′‐to‐3′ polarity of transcription for the major class of transcriptionally acting T‐boxes (Zhang & Landick, 2016), it is possible that by the time when Stem II emerges from the transcribing RNAP, tRNA is already bound to the long Stem I via bivalent interactions to the tRNA anticodon and elbow, the formation of which clearly do not require Stem II (Li et al., 2019). Subsequently, the late‐emerging Stem II engages the fully or partially formed codon–anticodon duplex stabilizing it. Alternatively, if tRNA binding is relatively slow compared to the rate of transcription and RNA folding, Stem II may have already been transcribed and folded, which then docks with Stem I prior to or concomitant with the arrival of tRNA. Detailed co‐transcriptional kinetic analyses, using rapid mixing, fluorescence, and single‐molecule methods are likely needed to distinguish among the possible scenarios. For translational T‐boxes that continuously experience reversible tRNA association and dissociation, the order of the binding events is also unknown and could be different from their transcriptional counterparts. As many translational T‐boxes lack the elbow‐binding Stem I IDTM, their truncated Stem I's do not seem to bind tRNA appreciably without Stem II, as evidenced by barely detectable affinity measured by calorimetry (Suddala & Zhang, 2019b). Specific to the translational T‐boxes, it is interesting to consider what happens to the T‐box conformation upon tRNA dissociation? Will Stems I and II remain docked, rapidly undock and stay undocked, or undergo rapid docking–undocking dynamics while waiting for another tRNA to bind? Addressing these open questions will require additional biophysical approaches and provide important insights into multilateral, multivalent, and sequential RNA–RNA interactions.

Third, there exist a large variety of lineage‐specific alterations to the core T‐box architecture. These features likely evolved in adaption to the diverse environmental conditions, nutritional profiles and regulatory needs of the microorganisms. There are fully and partially tandem T‐boxes whose modes of action remain unknown. There are curious insertions in every domain of the T‐box with unknown structure and function. The less conserved Stem II domain further harbors remarkable diversity in sequence and structure. These insertions may engage novel contacts with other regions of the tRNA. Given the large surface area and complex scaffold of tRNAs as compared to small‐molecule metabolites, it is not surprising that through evolution, the primordial T‐boxes have diverged and accrued a large collection of variations. Additionally or alternatively, some of these lineage‐specific elements may have evolved to permit crosstalk and coordination with other forms of RNA or ribonucleoprotein‐mediated gene regulation. For instance, a tandem arrangement of a T‐box and a ppGpp riboswitch was reported to form a Boolean “AND” gate (Sherlock, Sudarsan, Stav, & Breaker, 2018).

Finally, the recent elucidation of the core T‐box mechanisms has informed and continues to inspire rational design and engineering of RNA devices that can recognize the 3D structure of other RNAs or proteins, sense minute chemical modifications on them, catalyze desired chemical transformations on specific RNA targets, or assemble into programmed suprastructures using joints and interfaces ported from the T‐box paradigm. T‐boxes can serve as effective tools in synthetic biology thanks to their unique ability to decode specific tRNAs in the RNA form without translation. An outstanding example is the newly minted T‐box ribozyme named “Tx2.1”, which harnessed the tRNA‐binding specificity of the T‐box RNA to allow its flexizyme‐like moiety to aminoacylate specific tRNAs like their protein counterparts—the aminoacyl‐tRNA synthetases (aaRS; Ishida, Terasaka, Katoh, & Suga, 2020; Rubio Gomez & Ibba, 2020; Xiao et al., 2008).

## CONFLICT OF INTEREST

The author declares no conflicts of interest.

## RELATED WIREs ARTICLE

Structure and mechanism of the T‐box riboswitches
